# Research and Development of Self-Waterproofing Concrete for Tunnel Lining Structure and Its Impermeability and Crack Resistance Characteristics

**DOI:** 10.3390/ma16165557

**Published:** 2023-08-10

**Authors:** Huayun Li, Anxiang Zhou, Yangfan Wu, Lai Deng, Kaicheng Zhu, Feng Lu

**Affiliations:** 1 School of Architecture and Civil Engineering, Xihua University, Chengdu 610039, China; 2 School of Emergency Management, Xihua University, Chengdu 610039, China; 3 Key Laboratory of Transportation Tunnel Engineering, Ministry of Education, School of Civil Engineering, Southwest Jiaotong University, Chengdu 610031, China

**Keywords:** orthogonal design, mix design, microscopic testing, waterproof concrete, mechanical properties

## Abstract

This research paper systematically investigates the combined influence of fly ash, cementitious capillary crystalline waterproofing (CCCW) materials, and polypropylene fibers on the mechanical properties and impermeability of concrete through comprehensive orthogonal tests. Microscopic morphological changes in the concrete induced by different composite materials are examined via scanning electron microscopy (SEM) and X-ray diffraction (XRD) testing. The objective is to facilitate a beneficial synergetic interaction among these materials to develop highly permeable, crack-resistant concrete. Key findings of this study are: (1) The study unveils the impact of the concentration of three additive materials on the concrete’s compressive strength, tensile strength, and penetration height, thereby outlining their significant influence on the mechanical properties and impermeability of the concrete; (2) An integrated scoring method determined the optimal composite dosage of three materials: 15% fly ash, 2% CCCW, and polypropylene fibers at 1.5 kg/m^3^. This combination increased the concrete’s compressive strength by 12.5%, tensile strength by 48.4%, and decreased the average permeability height by 63.6%; (3) The collective introduction of these three materials notably augments the hydration reaction of the cement, resulting in denser concrete microstructure, enhanced bonding between fibers and matrix, and improved concrete strength and durability.

## 1. Introduction

As transportation networks continue to develop and expand, the frequency and scope of tunnel construction projects are increasingly growing. Concurrently, various issues, such as lining leakage, concrete cracking, falling debris, structural cracking, and damage due to freezing drainage systems, are persistently emerging. Of these, the common problem of tunnel lining leakage is the most prominent [1], and it is also a challenge encountered by traditional lining concrete. It has become one of the hot issues in tunnel engineering. Tunnel leakage not only jeopardizes driving safety but also undermines the efficiency of various infrastructural support systems within the tunnel. This leads to the corrosion of operational facilities, impacts the serviceability of the tunnel, and, in extreme cases, can result in casualties and economic losses [2]. In regions with complex geology and hydrology, corrosive ions can infiltrate the concrete lining with water, causing the corrosion of steel bars, which subsequently impairs the durability and load-bearing capacity of the lining structure [3]. As a result, it is crucial to focus on the impermeability and crack resistance of concrete during the construction process of tunnel engineering [4,5].

Currently, the primary method of tackling these issues involves designing an appropriate mix ratio, incorporating mineral admixtures and admixtures, which can enhance the impermeability and crack resistance of concrete structures. This approach aims to make the structure itself waterproof [6]. Structural self-waterproofing is largely dependent on the high mechanical and impermeability properties of the waterproof concrete itself, and its impermeability is determined by the compactness of the concrete’s internal structure. Addressing concrete’s impermeability necessitates a prior focus on cracking. The presence of macro and micro-cracks can reduce the compactness, impermeability, frost resistance, strength, and apparent density of concrete. Such cracks serve as the primary water seepage pathways within the concrete. Therefore, concrete’s crack resistance is a key determinant of the structure’s strength and durability, and controlling concrete cracking is crucial for enhancing the impermeability performance of the lining structure.

Several scholars have performed extensive research on the enhancement of concrete’s crack resistance by the addition of appropriate amounts of mineral admixtures to concrete mixtures. Zheng et al. [7] investigated the influence of fly ash content on the early cracking performance of High-Flowing Concrete (HFC). Their findings indicate that the cracking performance of HFC improves with an increasing amount of fly ash, provided the dosage is below the optimum level. Cheng et al. [8] conducted comparative experiments, XRD, and SEM analysis, determining that the optimal impermeability performance is achieved when 30% of cement is replaced with waste ceramic polishing powder. The impermeability of a mix with both waste ceramic polishing powder and fly ash outperforms that of mixes with only one of these additives. Omran, A, et al. [9] found that concrete mixed with biomass fly ash exhibited a denser microstructure and superior compressive, tensile, and bending strength. Liu et al. [10] found through an orthogonal test that the best working performance of concrete is achieved when the mineral content is 20%, sand ratio is 46%, stone powder content is 10%, and water–cement ratio is 0.30. However, the hydration reaction that occurs during the curing process of different mineral admixtures mixed into concrete often occurs as self-shrinkage [11,12,13]. Lee et al. [14] demonstrated that slag powder increases the autogenous shrinkage of concrete, with the shrinkage rate being directly proportional to the quantity of added slag powder. Shen et al. [15] studied the impact of adding 0%, 20%, 35%, and 50% of ground blast furnace slag to high-performance concrete during its early cracking performance using a temperature–stress testing machine. The results revealed that, as slag content increased, early temperature rise, cracking stress, and the cracking temperature of the concrete decreased, while autogenous shrinkage significantly increased, leading them to propose a reasonable slag content of 20%. Mazloom, M, et al. [16] observed that, when the replacement rate of the silica fume and cement increased from 6% to 15%, the 58-day autogenous shrinkage rate rose by 16.7–50% compared to the reference concrete. Akcay, B. et al. [17] found similar phenomena in their study on fiber self-compacting concrete. The influence of mineral admixtures on autogenous shrinkage and early cracking performance is primarily related to the admixtures’ activity effect [18] and morphological effect [19]. Admixtures with higher activity hasten the hydration process of cement, accelerate the water consumption rate in the mix, and increase capillary negative pressure, thereby enhancing the autogenous shrinkage rate and cracking potential. As such, it is concluded that fly ash, with its lower activity and circular particle shape, is particularly suitable for preparing waterproof concrete.

Adding mineral admixtures to concrete can trigger autogenous shrinkage, making it necessary to incorporate a specific amount of admixtures into the concrete to enhance its various properties. Numerous researchers have pursued extensive experimental and theoretical investigations into the impermeability and crack-resistant properties of fiber-reinforced concrete, yielding many significant results. Desmettre, C, et al. [20] probed the permeability of normal-strength concrete (NSC) and fiber-reinforced concrete (FRC) under a steady tensile load, employing innovative impermeability-testing apparatus. Their findings indicated that, under a static tensile load, FRC tension rod specimens exhibited 60–70% lower permeability than NSC tension rod specimens at the same stress level, underscoring the fibers’ beneficial contribution to the durability of reinforced concrete structures. According to a study by Yang et al. [21], high-performance concrete can be produced by combining 1.092 kg/m^3^ of polyvinyl alcohol fiber (PVA), 5 kg/m^3^ of imitation steel fiber (FST), and 32.8 kg/m^3^ of CSA expansive agent. This combination led to an 8.96% boost in compressive strength, a 28.2% rise in splitting tensile strength, and an 86.643% enhancement in impermeability. Liu et al. [22] found that adding polypropylene fibers ranging from 0 to 1.35 kg/m^3^ to high-performance concrete containing fly ash and slag powder could improve the concrete’s waterproofing capabilities and increase polypropylene content. Zhang et al. [23] explored the impact of polypropylene fibers on the mechanical properties of fly ash and silica fume concrete. Although the addition of fibers decreased the compressive strength of the concrete with 15% fly ash and 3% silica fume, it increased the splitting tensile strength of the concrete and significantly enhanced its ductility. Wang et al. [24] compared the impermeability performance of basalt fiber-reinforced concrete with polypropylene fiber-reinforced concrete. Their research indicated that adding these two types of fibers to standard concrete could decrease the concrete’s permeability height. Polypropylene fibers performed better than basalt fibers, and longer fibers outperformed short-cut fibers. When the length of the polypropylene fibers was 12 mm and the dosage was 0.9 kg/m^3^, the permeability height of the concrete was reduced by 59.44% compared to standard concrete. In addition, numerous researchers have investigated the effect of adding permeable crystalline waterproof material on the impermeability and mechanical properties of concrete. For instance, Zheng et al. [25] introduced a Penetron Admix (PA) to cement paste and studied its influence on cement paste’s impermeability. They found that, when the PA content was 1.6%, the permeability coefficient of the cement paste dropped by 93.2%. Joa et al. [26] added a 3% crystalline admixture to mortar and analyzed its impact on the physical and mechanical properties of the mortar. Their experimental results demonstrated that the addition of crystalline admixtures could substantially improve the mortar’s mechanical properties while reducing its porosity and water absorption [27].

In conclusion, numerous researchers have confirmed the beneficial effects of various concrete-reinforcing materials [28,29,30,31], analyzing and elaborating on the reinforcement materials’ mechanisms based on extensive experimentation. The previous research has laid a solid foundation for the further studying of the impermeability of concrete [32,33] and has achieved good results in the development of self-waterproofing concrete [34], such as the addition of waterproofing admixtures [35] and crushed ceramic jars [36] in concrete. However, previous studies have mainly focused on the addition of admixtures, but there are still many shortcomings, such as relatively few studies on the co-blending of different types of admixtures. Therefore, in this paper, an orthogonal design of the experiments’ scheme is applied to mix polypropylene fiber and fly ash with a permeable crystalline waterproofing agent capable of crystallizing in water and self-repairing cracks. This approach aims to exploit the synergistic benefits of different materials to create highly permeable, crack-resistant concrete, offering a potential solution to the issue of tunnel leakage.

## 2. Benchmark Mix Design

Before employing the orthogonal design, it is essential first to devise the mix proportion of the benchmark concrete. Based on the benchmark mix proportion, the cement consumption is replaced by an equivalent quantity of fly ash and cementitious capillary crystalline waterproofing materials, ensuring the water–cement ratio remains consistent across all test piece groups. The benchmark concrete can also act as a blank control during the experiment, used to gauge the modification effect of each group of the samples in the orthogonal experiment. By calculating the benchmark mix ratio and carrying out trial mixing, the workability and mechanical properties of the concrete are ensured, forming a critical foundation for the entire experiment.

### 2.1. Test Raw Materials and Performance Indicators

The cement utilized for concrete pouring is P·O 42.5 ordinary Portland cement, and its physical performance indicators are detailed in Table 1. The coarse aggregate is continuously graded crushed stone with a nominal diameter of 5–16 mm, and the fine aggregate comprises machine-made sand with continuous grading and a fineness modulus of 2.6. Class I fly ash is used as the mineral admixture, and the cementitious capillary crystalline waterproofing material is an XYPEX waterproofing agent, the performance indicators of which are listed in Table 2. Polypropylene fiber of 19 mm length is used in the experiment, and its performance indicators are listed in Table 3.

### 2.2. Calculation of Benchmark Mix Proportion and Indoor Trial Mixing Test

Tunnel secondary lining concrete possesses unique attributes, including low strength, high fluidity, and superior impermeability [37]. Unlike concrete used in other structures, the concrete used for secondary lining does not necessitate high strength, as the bulk of the tunnel load is predominantly borne by the initial support, with the secondary lining acting principally as a safety reserve. Therefore, this research conducted a mixed design for concrete with a strength grade of C35 [38]. Due to the constraints of the tunnel construction environment, executing concrete vibration work is challenging, necessitating high workability standards for the concrete mixtures. Therefore, when calculating the mix ratio, it is crucial to prioritize controlling the concrete’s workability, alongside ensuring strength, to comply with construction requirements. As per the specification for the mix proportion design of ordinary concrete JGJ55-2011 [39], the mix ratio was computed, and the calculation results are presented in Table 4.

Owing to variations in the properties of raw materials, the mix proportion computed as per the specifications cannot be directly utilized for specimen preparation. It is crucial to conduct trial mixing for the calculated mix ratio, determining the final trial mix ratio to guarantee the concrete mixture’s workability and fundamental mechanical properties.

**Figure 1:**
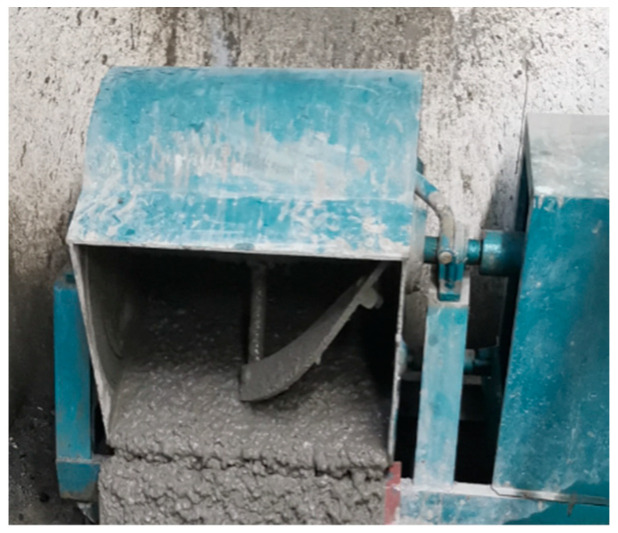
Forced mixer.

**Figure 2:**
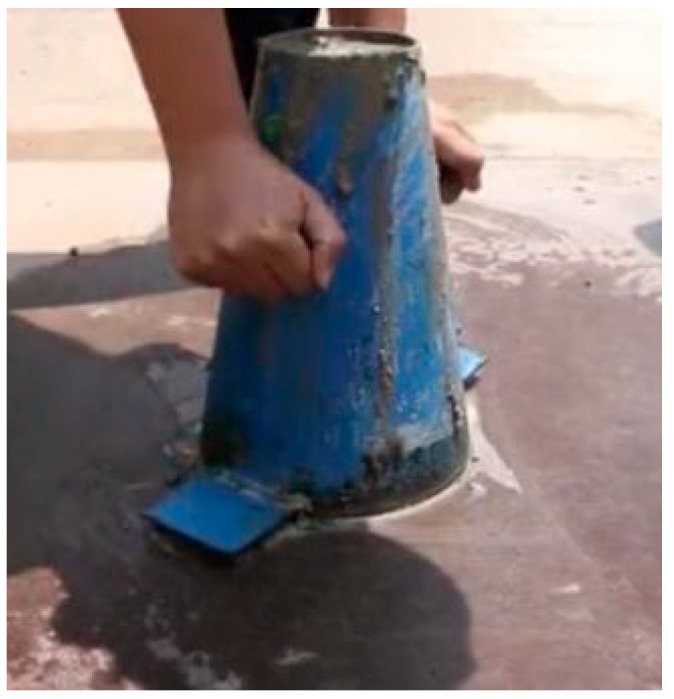
Slump test.

**Figure 3:**
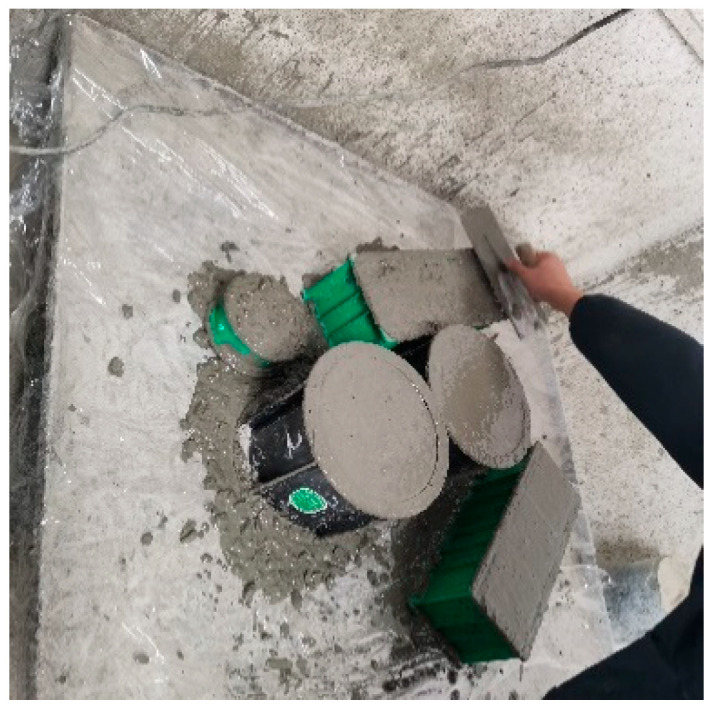
Vibration table.

A forced mixer with a 50 L capacity is used for concrete mixing, as illustrated in Figure 1. The mixing process is outlined as follows: cement and fine aggregate are initially added to the mixer and mixed for 2 min. Water is then introduced to form cement mortar with the fine aggregates. Subsequently, crushed stones are added and mixed for 4 min to ensure that the cement mortar fully wraps the stones. Lastly, a water-reducing agent is incorporated to fine-tune the mixture’s fluidity. Upon completion of the mixing, the mixture is poured out for bleeding observation. A slump test is then promptly performed, as depicted in Figure 2. Post-experiment, the concrete is molded into shape, as demonstrated in Figure 3. Once the concrete has fully hardened, it is demolded and transferred to a standard curing box for curing. Compressive strength tests are undertaken after 7 days to evaluate the early strength of the concrete.

Through trial mixing, it was discovered that the concrete prepared according to the calculated mix ratio exhibited bleeding, necessitating adjustments to the calculated mix ratio. The first approach was to reduce the water–cement ratio while maintaining the same dosage of cementitious materials. However, it was observed that a decrease in water usage significantly impacts the concrete’s fluidity, yielding a slump value of only about 94 mm. The second approach was to increase the sand ratio, consequently increasing the concrete’s specific surface area, which, in turn, raises water demand and reduces free water content, all while maintaining the same slump. Through trial mixing, it was discovered that, after increasing the sand rate by 2%, the concrete mixture has good workability, and that the 7-day compressive strength was 32.3 MPa, satisfying the early strength and workability requirements of tunnel-lining concrete. Thus, the test mix of benchmark concrete can be established, as shown in Table 5.

## 3. Research on the Significance of Factors Influencing the Permeability and Crack Resistance of Concrete Based on Orthogonal Design

### 3.1. Design of Orthogonal Experimental Scheme

Before undertaking the orthogonal design of experiments, it is essential to first define the purpose of the test, proposing test indicators to evaluate the quality of the test results for this purpose. This experiment’s objective is to simultaneously add different materials to concrete, enhancing the concrete’s impermeability and crack resistance for the secondary lining structure of tunnels. Three test indicators, namely, concrete compressive strength, splitting tensile strength, and penetration height, are proposed for this experimental purpose. Compressive strength ensures the lining structure’s load-bearing capacity, splitting tensile strength is a standard indicator for evaluating concrete’s crack resistance performance, and the penetration height of concrete can reflect its ability to resist water penetration.

Upon determining the experimental indicators, the factors that may influence the experimental results and establish the corresponding levels are selected. Factor selection often requires a certain amount of practical experience and literature support. Through comprehensive analysis and a summary of existing research findings, this article selects fly ash as a representative material of mineral admixtures, polypropylene fibers as a representative material of fibers, and cementitious capillary crystalline waterproofing materials as a representative material of additives. These are selected as three factors to improve the concrete’s impermeability and crack resistance. Based on an exhaustive review of the pertinent literature, the corresponding levels of these three factors are established as shown in Table 6.

After determining the factors and levels of the orthogonal experiments, an appropriate orthogonal table can be selected for meter design. The orthogonal table should neither be too large nor too small. An overly large orthogonal table not only fails to significantly contribute to the experimental results but also impacts work efficiency, while a small orthogonal table may affect the experiment’s accuracy. Given that three factors and levels are involved in this experiment, the L_9_(3^4^) orthogonal table is the most sensible choice for arranging the experiment. The mix ratio of concrete specimens is based on the C35 test mix ratio. To maintain a constant water–binder ratio, fly ash and permeable crystalline waterproofing agents are added to the mixture in equal amounts to replace the cement. The final mix proportions of nine sets of specimens and the benchmark concrete for the orthogonal experiment are shown in Table 7.

### 3.2. Laboratory Test of Concrete Mechanics and Impermeability

The pouring of concrete specimens also utilizes a forced mixer. Before pouring, the weight of each part of the material should be accurately measured, and the mixing mechanism is similar to that of the benchmark concrete. That is, the cementitious material, sand, and polypropylene fibers are first put into the mixer for dry mixing for 2 min to evenly distribute the fibers and avoid agglomeration; then, water is added and mixed for another 2 min to form cement mortar. Afterward, crushed stone and water-reducing agent are added and mixed thoroughly for 2 more minutes. After mixing, the mixture is poured into a mold, compacted using vibrating, and allowed to stand for 24 h. Then, the mold is removed, labeled with a number, and placed in a standard curing box for 28 days of curing. The mechanical properties were tested using 100 mm × 100 mm × 100 mm cubic specimens, and impermeability performance was tested using standard trapezoidal specimens with dimensions of 185 mm × 175 mm × 150 mm.

#### 3.2.1. Concrete Compressive Strength Test

**Figure 4:**
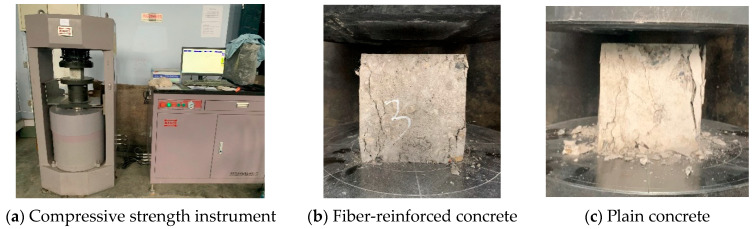
Compressive strength test.

After 28 days of curing the concrete specimens, mechanical performance tests should be conducted promptly, and the test method should refer to the Standard for test methods of concrete physical and mechanical properties GB/T50081-2019 [40]. The pressure machine used in this experiment is shown in Figure 4a, with a loading rate of 0.5 MPa/s. The failure state of the specimen is shown in Figure 4b,c. Plain concrete typically exhibits brittle failure, with severe peeling of the specimen surface. However, after fiber concrete is crushed, it can maintain some residual strength as a whole. This is because polypropylene fibers can have a good tensile effect when concrete cracks under compression, preventing it from experiencing significant brittle failure.

Three concrete specimens with the same number form a group, and the average value is taken as the final compressive strength value. Since the specimens used in this experiment are non-standard specimens, the average compressive strength value should also be multiplied by a reduction factor of 0.95. The specific calculation method is carried out according to Equation (1).

(1) $$f_{cc}=\frac{F}{A}$$

Among them$,{\;f}_{cc}\;$ is the compressive strength of concrete cube specimens (MPa); F is the failure load of the specimen (N); A is the pressure bearing area of the specimen (mm^2^).

#### 3.2.2. Concrete Splitting Tensile Strength Test

**Figure 5:**
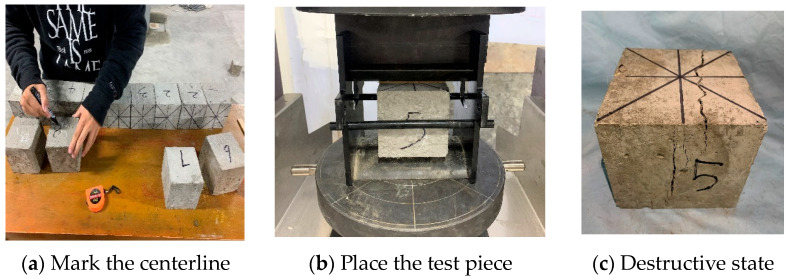
Splitting tensile strength test.

The tensile strength of concrete plays a significant role in its anti-cracking performance. In structural design, tensile strength is an important indicator for assessing the crack resistance performance of concrete, and it is also employed to evaluate the bonding strength between the concrete matrix and steel bars. The splitting tensile strength test is often used to determine the tensile strength of concrete. The “splitting tensile strength test” program in the press control system mentioned above is employed for the test, and the test equipment includes steel arc-shaped cushion blocks, plywood cushion strips, and straightedges. For C35 concrete, the loading rate should be between 0.05 MPa/s and 0.08 MPa/s [40], and 0.05 MPa/s was chosen for this test. The experimental process is depicted in Figure 5a–c.

The tensile strength value of concrete is also taken as the average of three specimens, and, for non-standard specimens, a reduction coefficient of 0.95 should be taken. The calculation method should be carried out according to Equation (2):

(2) $$f_{ts}=\frac{2F}{\pi A}=0.637\frac{F}{A}$$

Among them, $f_{ts}\;$ is the splitting tensile strength value (MPa); F is the ultimate load (N); A is the splitting surface area (mm^2^).

#### 3.2.3. Concrete Impermeability Performance Test

This study uses a YC-HS4.0 intelligent concrete impermeability tester to measure and conduct experimental research on impermeability performance. This instrument has a maximum allowable working pressure of 4.0 MPa, and the pressurization method is automatic. Six specimens can be tested at once. The steel mold size is 175 mm in diameter at the upper mouth, 185 mm in diameter at the lower mouth, and 150 mm in height, complying with the requirements for the number and size of specimens in the Standard for the test methods of the long-term performance and durability of ordinary concrete GB/T50082-2009 [41].

**Figure 6:**
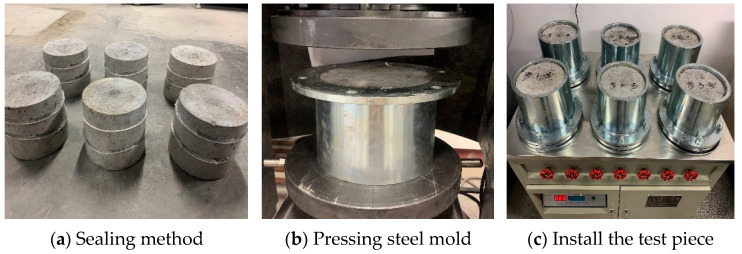
Concrete impermeability test.

When using the penetration height method for testing, first, remove the specimen from the curing box and wipe it clean. Then, seal the specimen. Due to environmental concerns and the operational complexity associated with using paraffin as a sealing method, an alternative approach using rubber rings and glass glue was utilized in this study. Firstly, the rubber ring is placed at the upper and lower positions of the test piece. Then, glass glue is applied to the position of the rubber ring. Finally, use a press to press the test piece into the steel mold and let it sit for 12 h to complete the sealing. The operation process is shown in Figure 6a–c. The practical results show that this sealing method is not only easy to operate but also offers good sealing performance, reducing the phenomenon of water leakage from the side of the steel mold and improving the success rate of the experiment.

**Figure 7:**
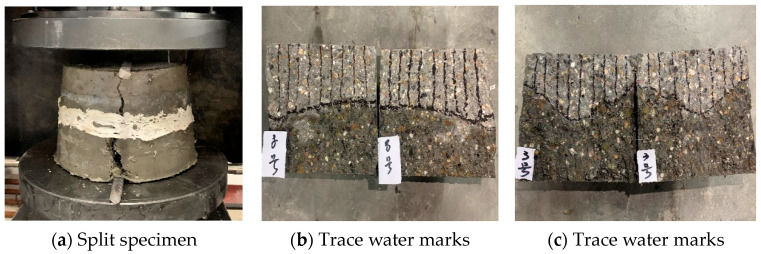
Penetration height testing process.

After the sealing is done, the testing can commence. Before installing the test piece, open the six water valves to fill the test tank with water, hence discharging the gas from the water pipe and test tank. During the installation of the test piece, ensure the screws are tightened properly to maintain the sealing of the entire instrument. At the beginning of the test, set the water pressure to 1.2 MPa and observe if there is any seepage around the specimen. If seepage is observed, the specimen should be removed and resealed. Close the outlet valve after a set of six specimens have undergone 24 h of exposure to 1.2 MPa water pressure, hence concluding the test. Subsequently, use a press to split the penetrated concrete specimen along its centerline, mark the water stains with a waterproof pen, and evenly divide the water stains into ten equal parts. Then, measure the penetration height and calculate the average value. The operation process is shown in Figure 7a–c.

The calculation of permeability height is carried out according to Equations (3) and (4):

(3) $${\overset{-}{h}}_{i}=\frac{1}{10}\sum_{j=1}^{10}h_{j}$$

(4) $$\overset{-}{h}=\frac{1}{6}\sum_{i=1}^{6}\overset{-}{h_{i}}$$

In the equation, $h_{j}$ is the penetration height value of the j-th measuring point of the i-th specimen. ${\bar{h}}_{i}$ is the average penetration height of the 10th measuring point of the i-th specimen. $\bar{h}$ is the average value of the water seepage height of a group of specimens. The unit is mm, which is the final test result of the penetration height of a group of specimens.

After calculating the penetration height of each group of specimens, the relative permeability coefficient can be determined based on the water pressure and penetration time. This coefficient represents the impermeability of the concrete. The smaller the relative permeability coefficient, the stronger the concrete’s ability to resist liquid penetration. The calculation method is as follows [41]:

(5) $$S_{K}=\frac{mD_{m}^{2}}{2TH}$$

Among them, $S_{K}\;$ is the relative permeability coefficient (mm/s); m is the water absorption rate of concrete, generally taken as 0.03; $D_{m}$ is the average penetration height of the specimen (mm); T is the penetration time (s); H is the water pressure, represented by the height of the water head—a water pressure of 1 MPa is equivalent to 102,000 mm.

### 3.3. Calculation and Analysis of Orthogonal Test Results

The beauty of orthogonal experiments lies in their ability to carry out a comprehensive analysis using a minimal number of tests, thereby revealing the significant impact of various factors on the experiment indicators when they interact. The methods of analysis encompass range analysis and variance analysis.

#### 3.3.1. Compressive Strength Test Results and Analysis

**Figure 8:**
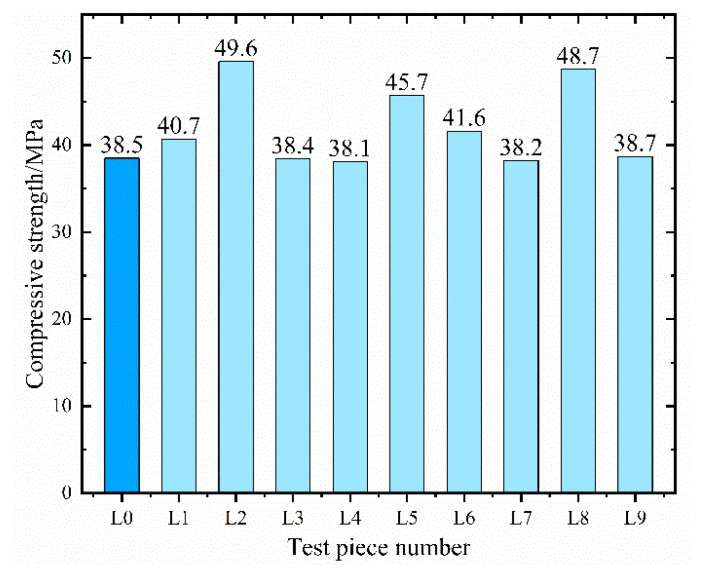
Twenty-eight-day compressive strength test results.

Figure 8 showcases the strength values of ten groups of concrete samples as measured by the compressive strength test.

Compared to conventional C35 concrete, the compressive strength of concrete mixed with modified materials typically produces an enhancement. Notably, the strength of L2 concrete exhibits the highest surge of 28.8%, suggesting that the amalgamation of fly ash, polypropylene fiber, and CCCW contributes positively to the growth of concrete strength. Nevertheless, in the realm of orthogonal experiments, a mere comparison of each group of samples is not sufficient. It becomes imperative to perform range analysis and variance analysis on the experimental outcomes to decipher their inherent patterns of influence. Table 8 displays the outcomes of the range analysis.

**Figure 9:**
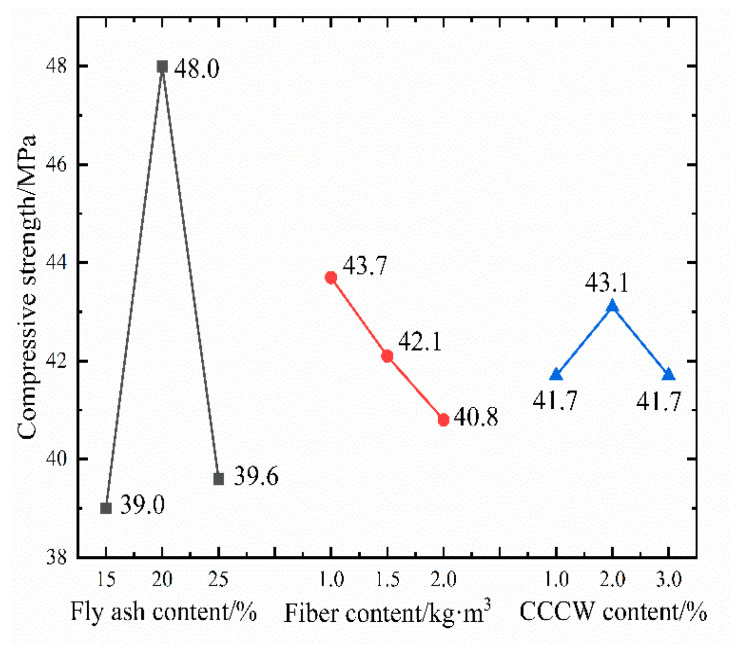
Trend of compressive strength variation.

When it comes to compressive strength, the three factors’ impact on the test results is primarily and secondarily ordered as A > B > C. This implies that the quantity of fly ash has the most substantial effect on the strength of the concrete, while the influences of polypropylene fiber and CCCW on compressive strength are not significant. The strength enhancement of concrete due to fly ash can be attributed to three factors: the volcanic ash effect [42], the micro aggregate effect [43], and the morphological effect [44]. The volcanic ash effect suggests that the active substances in fly ash react with the Ca(OH)_2_ produced by the cement hydration reaction, generating denser hydrated calcium silicate and hydrated calcium aluminates, thereby elevating the structural strength. Moreover, the diminutive particles of fly ash augment the aggregate’s specific surface area, enabling the mix to have sufficient mortar to fill the pores between the coarse and fine aggregates. This plays a lubricating role, enhancing the mix’s workability and ultimately increasing the concrete’s strength, a phenomenon referred to as the micro aggregate effect of fly ash. The morphological effect alludes to the fact that most fly ash particles are spherical, with a smooth surface and minimal pores. This structure decreases the mix’s water demand, heightens its fluidity, and facilitates easier compaction of the concrete. It was found that the addition of fiber [45], shrinkage-reducing agent (SRA), and superabsorbent polymer (SAP) [46] to concrete improved the compressive strength of coagulation, but the combination of fly ash, polypropylene fiber, and CCCW also showed good compatibility, and the compressive strength was also well strengthened. The optimal dosage of the three can be analyzed from the indicator factor diagram, shown in Figure 9. The significance of the three factors’ impact on compressive strength can be ascertained through variance analysis, as depicted in Table 9.

The calculation reveals that the *p*-value of factor A is less than 0.05, indicating that the amount of fly ash has a notable impact on the concrete’s compressive strength. The inclusion of polypropylene fiber slightly impacts the compressive strength, whereas CCCW exerts a minimal effect on the concrete’s compressive strength. To summarize, for the compressive strength index, the optimal composite dosage of the three is A_2_B_1_C_2_. Verification experiments established that the average compressive strength of concrete with a factor level combination of A_2_B_1_C_2_ was 49.8 MPa, substantially enhancing the compressive bearing capacity of ordinary concrete.

#### 3.3.2. Splitting Tensile Strength Test Results and Analysis

**Figure 10:**
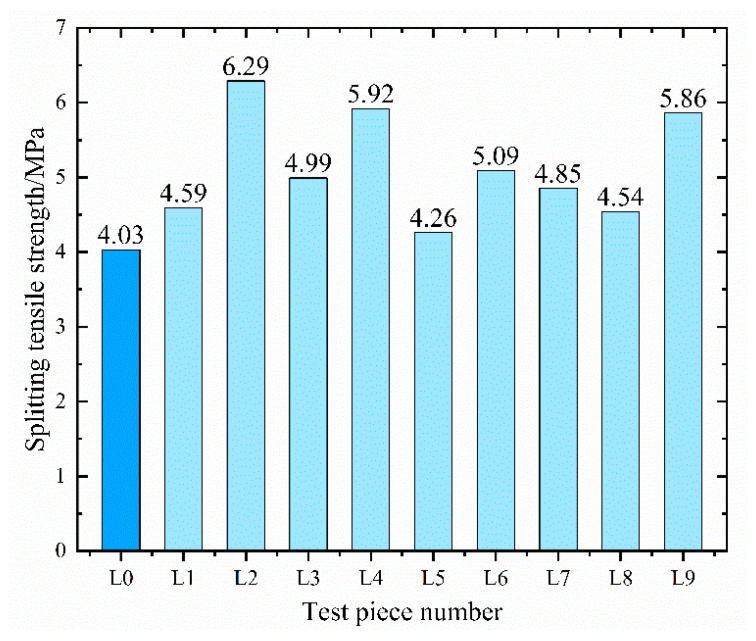
Twenty-eight-day splitting tensile strength results.

Figure 10 displays the tensile strength values of ten sets of specimens as determined using the splitting tensile strength test.

The tensile strength of the benchmark concrete stands at 4.03 MPa, which represents the lowest value among the ten sets of specimens. The two sets of specimens with the highest tensile strength, L2 and L4, display values of 6.29 MPa and 5.92 MPa, respectively. In comparison to the benchmark concrete, their growth rates are 56% and 47%, respectively. Generally, the addition of fly ash, fibers, and waterproofing agents appears to enhance the concrete’s crack resistance performance. However, to delve into the three factors’ impact on tensile strength and ascertain the optimal dosage, it is imperative to conduct range analysis and variance analysis on the test results. Table 10 and Table 11 present the analyses’ results.

The calculations suggest that the dosage of polypropylene fibers is the paramount factor influencing the concrete’s tensile strength, followed by CCCW. Conversely, fly ash does not have a significant effect on bolstering the concrete’s tensile strength. Unlike mineral admixtures, polypropylene fibers mainly modify concrete through physical processes. Once sufficiently mixed and subjected to friction, the fiber bundles disperse into thousands of individual fibers, uniformly distributed within the concrete, thereby forming a fiber network structure. This structure provides a significant “supporting” role [47], resulting in a more uniform particle distribution. Although this may somewhat curtail the mixture’s fluidity, it leads to a denser concrete structure. Furthermore, polypropylene fibers can considerably minimize microcracks caused by early plastic shrinkage and temperature stress and reduce stress concentration at the ends of the cracks, hence inhibiting crack development [48].

**Figure 11:**
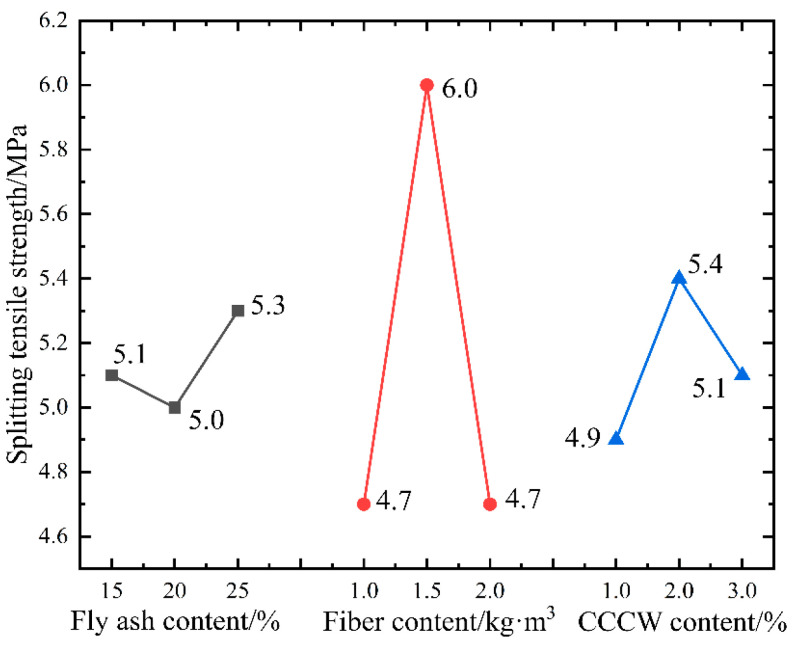
Change trend of splitting tensile strength.

The variance calculations indicate that factor B’s *p*-value is 0.02 < 0.05, statistically highly significant, suggesting that polypropylene fibers indeed play a crucial role in enhancing the tensile strength of the concrete. The *p*-value of factor C lies between 0.1 and 0.2, suggesting that permeable crystalline waterproofing agents also influence the concrete’s crack resistance performance to some extent. Further investigation is required to establish whether the two exhibit a “positive hybrid effect”, meaning whether the addition of waterproofing agents boosts the adhesion between the fibers and the matrix. Figure 11 displays the influence of the horizontal changes of the three factors on tensile strength.

The alteration in fly ash content does not induce significant fluctuations in the concrete’s tensile strength. Within a range of 15–25% of content, the maximum tensile strength fluctuation is merely 0.3 MPa. As the polypropylene fiber content increases, the tensile strength initially rises before descending. This is because an appropriate amount of fibers can, under the action of a mixer, form individual fibers that are evenly dispersed within the coarse and fine aggregates and mortar, thus enabling them to resist plastic shrinkage and cracking. The addition of excessive fibers not only curtails the mixture’s fluidity, but also leads to agglomeration, creating too many defects inside the concrete and significantly impairing its performance. This phenomenon is more likely to occur when the fibers have a larger aspect ratio. The experimental findings indicate that, when the dosage of polypropylene fiber is 1.5 kg/m^3^, the maximum tensile strength attained is 6.0 MPa. The tensile strength increases with the content of permeable crystalline waterproofing agents until it reaches 2% of the cementitious material, after which it starts to decrease. This decrease is because excessive CCCW can impact the hydration of the cement, leading to a reduction in the generated amount of hydrated calcium silicate cement, thus affecting the concrete’s crack resistance [49]. To summarize, for the splitting tensile strength indicator, the optimal composite dosage of the three factors is A_3_B_2_C_2_. Subsequent validation tests found that the concrete’s average tensile strength was 6.31 MPa with a combination of factor levels of A_3_B_2_C_2_. This represents a 56% increase in tensile strength compared to the benchmark concrete, enhancing the concrete’s crack resistance.

#### 3.3.3. Results and Analysis of Impermeability Performance Test

**Figure 12:**
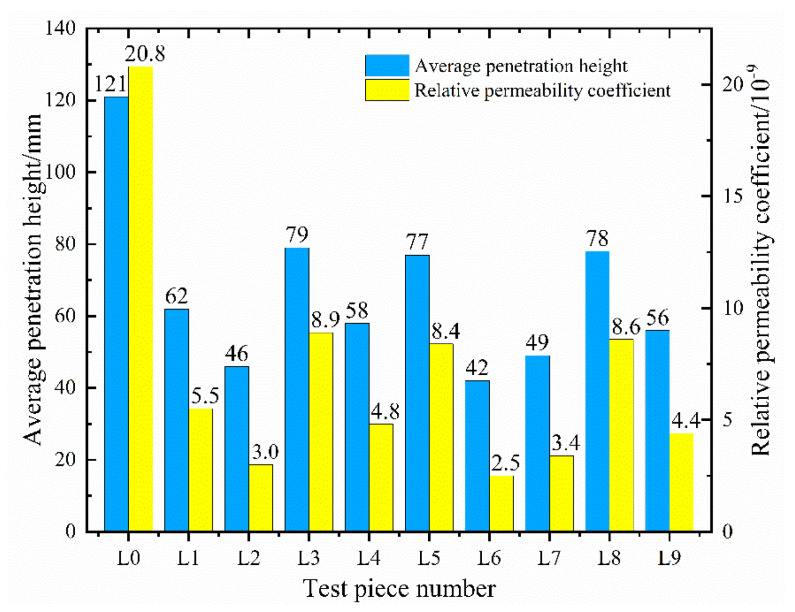
Permeability height and relative permeability coefficient.

The concrete impermeability test specimens are divided into six groups, and the average value of the water permeability height is taken as the permeability height of this group of specimens. Through statistical analysis and the organization of the test results, the permeability height and relative permeability coefficient of ten groups of specimens are obtained, as shown in Figure 12.

The test results indicate that the permeability height of ordinary C35 concrete is 121 mm, and the relative permeability coefficient is 2.08 × 10^−8^ mm/s. The inclusion of fly ash, polypropylene fiber, and permeable crystalline waterproofing agent in concrete in specific proportions results in a significant decrease in their permeability height, thereby significantly enhancing the concrete’s impermeability. The most substantial reduction occurs in L6, which has a water permeability height of approximately 1/3 of that of the reference concrete and an 88% reduction in the relative permeability coefficient. The smallest reduction is in L3, but its relative permeability coefficient is only 43% of the benchmark concrete. Overall, the addition of the three materials significantly improved the concrete’s impermeability performance. However, to optimize the mix ratio and analyze the internal influencing laws, range analysis and variance analysis are also needed, as presented in Table 12 and Table 13.

**Figure 13:**
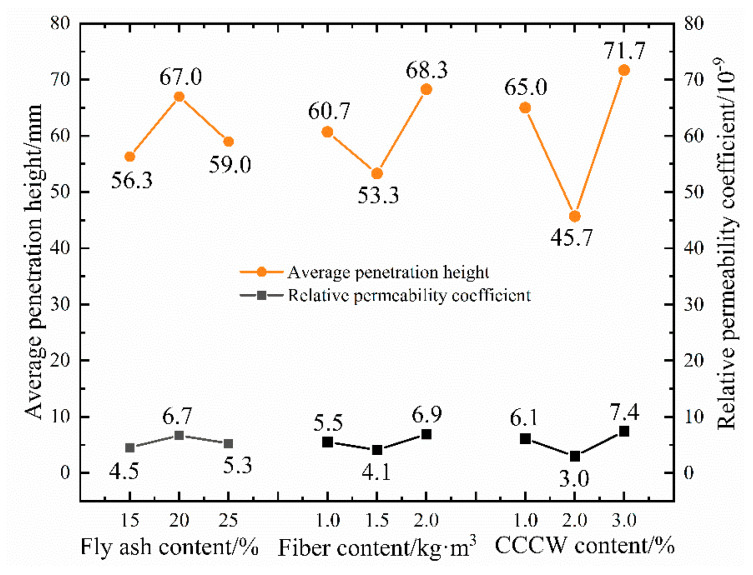
Change trend of permeability height and permeability coefficient.

Through range analysis, we obtain the order of the three influencing factors as R_C_ > R_B_ > R_A_. This suggests that CCCW is the most crucial factor affecting concrete’s impermeability, followed by the quantity of polypropylene fiber added, and, finally, the presence of fly ash. The *p*-values of factors B and C in the variance analysis are 0.05 and 0.02, respectively, demonstrating a significant impact of polypropylene fibers and CCCW on the impermeability of concrete. Furthermore, factor A has a *p*-value of 0.08, which, although greater than 0.05, is less than 0.1, implying that fly ash also contributes to improving the impermeability of concrete. Even though all three materials enhance the impermeability of concrete, their action mechanisms are distinct. Currently, the action mechanism of CCCW is broadly understood to be the “precipitation mechanism” or the “complexation precipitation mechanism” [49,50,51]. Upon introducing cementitious capillary crystalline waterproofing materials to fresh concrete, the active chemical substances in these materials seep into the concrete via the mixture’s water, react with free Ca^2+^ and oxides, generate insoluble crystals, and seal the pores and microcracks in the concrete. This process aids in waterproofing, enhancing the concrete’s resistance to water permeability. Polypropylene fibers improve the impermeability of concrete by minimizing defects during its hardening process, specifically by controlling the generation of shrinkage cracks and segregation cracks, preventing the formation of through-cracks inside the concrete [52,53]. Conversely, fly ash increases the compactness of concrete by providing a shape effect, micro aggregate effect, and volcanic ash effect. This decreases the porosity between the coarse aggregate and cement matrix, thereby enhancing the concrete’s waterproof performance. The variation trend of permeability height and relative permeability coefficient with the level of factors is shown in Figure 13.

When increasing the fly ash content from 15% to 25% of the cementitious material, the penetration height initially increases and then decreases. The corresponding penetration heights at the three levels reduce by 53%, 45%, and 51% compared to the benchmark concrete, with the optimal content being 15%. Increasing fiber content causes the permeability height to first decrease and then increase, demonstrating yet again that excessive fiber content negatively impacts the internal compactness of the concrete. The permeability height corresponding to the three types of polypropylene fiber content is reduced by 50%, 56%, and 44% compared to ordinary C35 concrete, with an optimal content of 1.5 kg/m^3^. The permeability height’s changing trend with the lateral change of CW is akin to that of polypropylene fiber, which first decreases and then increases. As the proportion increases, the permeability height decreases by 46%, 62%, and 41%, respectively. The optimal proportion is 2% of the cementitious material. The trend of the relative permeability coefficient changes aligns with the permeability height. Thus, for the penetration height indicator, the optimal combination of the three factors is A_1_B_2_C_2_.

#### 3.3.4. Comprehensive Scoring of Multiple Indicator Problems

The previous analysis considered a single indicator, and the optimal factor level combination obtained corresponded to a single indicator. Clearly, it did not fulfill the objective of this study, which is to prepare concrete with both high impermeability and high cracking resistance. Consequently, a comprehensive scoring method was employed to convert multiple indicators into a singular indicator. This method involves assigning different importance coefficients to each indicator based on the experimental purpose and each indicator’s independent analysis results. The product of each indicator’s experimental results and their importance coefficients are then added to calculate the comprehensive score of each combination. This enables the use of a single indicator analysis method to derive conclusions from a multi-indicator experiment [54]. The key to the comprehensive scoring method is determining the importance coefficients of each indicator appropriately, which demands judgments based on experimental goals and practical experience. As of now, there exists no general mathematical formula for this.

In this orthogonal experiment, the significance of the concrete’s compressive strength is relatively low. This is because the tunnel’s secondary lining structure mainly serves as a safety reserve, necessitating that its compressive strength merely meets the design requirements. However, both tensile strength and impermeability performance relate to the concrete’s durability, the key research objective of this study; therefore, their importance is relatively high. By taking various influencing factors into consideration, and based on the analysis results of different independent indicators, we can define the following score calculation method for each combination:

Comprehensive Score=(150−Penetration Height) × 2.5+Splitting Tensile Strength × 2.5+Compressive Strength × 0.5

Here, “150-penetration height” refers to the height of the impermeable specimen that remains unpenetrated by water. This calculation aims to ensure that a higher comprehensive score reflects the concrete’s superior comprehensive performance. Subsequent calculation provides the range analysis and analysis of variance for the comprehensive scores, as shown in Table 14 and Table 15.

The extreme differences in the comprehensive scores for the three test indices suggest that the primary factors affecting the mechanical properties and durability of the concrete, in descending order, are C > B > A. The optimal factor level combination is A_1_B_2_C_2_. The variance analysis results indicate that the significance probabilities *p* of factor B and factor C are both less than 0.05, demonstrating that the addition of composite fibers and permeable crystalline waterproofing agents significantly impacts the mechanical and impermeable properties of the concrete. The optimal factor level combination obtained from both the single indicator analysis and multi-indicator analysis were simultaneously used for validation experiments, the results of which are shown in Table 16.

Verification testing shows that the optimal combination of factor levels obtained through an analysis of individual indicators does indeed lead to the highest value of the corresponding indicator. When compared to the benchmark concrete, the A_2_B_1_C_2_ concrete displays the highest compressive strength improvement rate at 29.3%, and the A_3_B_2_C_2_ concrete has the highest tensile strength improvement rate at 56%. The concrete with a factor level combination of A_2_B_1_C_2_ is not only the optimal solution considering the single indicator of permeability height but also the optimal solution for a comprehensive evaluation. The verification test results indicate that the A_1_B_2_C_2_ concrete achieves the highest comprehensive score, its compressive strength meets the design requirements of tunnel engineering, and its tensile strength and permeability height are also at optimal levels. This greatly enhances the concrete’s resistance to cracking and permeability.

## 4. Microscopic Mechanism Analysis of the Effect of Composite Modified Materials on the Properties of Concrete

The micro-morphological characteristics and material composition of concrete often dictate its macroscopic properties. Prior tests on mechanical properties and impermeability indicated that the inclusion of three modified materials significantly enhances the macroscopic properties of concrete. To further investigate the micro-mechanism of fly ash, polypropylene fiber, and cementitious capillary crystalline waterproofing materials during the hydration and hardening process of concrete, we performed scanning electron microscopy experiments and X-ray diffraction quantitative phase analysis experiments on concrete mixed with varying modified materials aged at 28 days. Through a comparative analysis, we aim to explain the modification mechanism of external materials and the interaction effect between these materials from a microstructural standpoint.

### 4.1. SEM Scanning Electron Microscopy Analysis

#### 4.1.1. SEM Scanning Electron Microscope Test Plan

In order to study the influence of different additive materials on the microstructural characteristics of concrete [55], we prepared four groups of concrete for this study. These groups are as follows: solo polypropylene fiber concrete, a combination of polypropylene fiber and fly ash concrete, a combination of polypropylene fiber and CCCW concrete, and a combination of polypropylene fiber, fly ash, and CCCW concrete. Given that polypropylene fibers primarily play a physical role within the concrete and do not impact the cement’s hydration products and crystallization, this experimental design allows us to not only compare the differences in the microstructure of the concrete matrix caused by different cementitious composite systems but also to investigate the bonding characteristics at the fiber–matrix interface. Based on prior research results, the dosage of polypropylene fiber is set at 1.5 kg/m^3^, the dosage of fly ash is set at 15% of the cementitious material, and the dosage of CCCW is set at 2.0% of the cementitious material. The mixing proportions and specimen numbers for the four groups of concrete are shown in Table 17.

#### 4.1.2. SEM Scanning Electron Microscope Test Results and Analysis

We observed four groups of concrete samples using a field emission scanning electron microscope (Thermo Scientific APREO 2C), focusing on the micromorphology of the concrete matrix and the bonding characteristics between polypropylene fibers and the matrix. We divided the observation results into the following categories for analysis:

(1) Microscopic Morphological Characteristics of Concrete Matrix at 28 Days of Age

**Figure 14:**
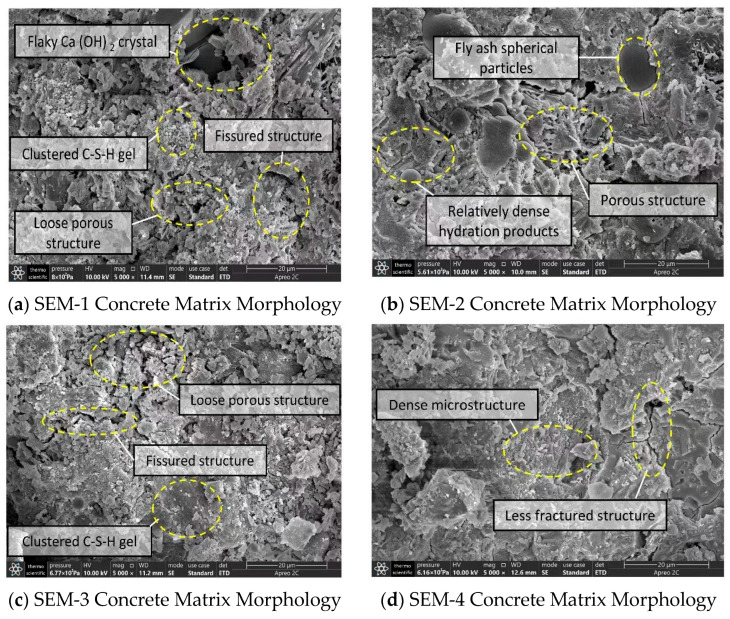
Microscopic morphological characteristics of concrete matrix at 28 d age magnified by 5000 times.

Figure 14a–d presents scanning electron microscope images of the apparent morphological characteristics of the four concrete groups after 28 days of hydration reaction, all magnified by 5000 times. As shown, different cementitious composite systems display significant microstructural differences post-hydration. The cementitious material in SEM-1 concrete is cement, and thus the crystal shown in Figure 14a is a cement hydration reaction product. Lamellar Ca(OH)_2_ crystals, abundant clustered calcium silicate hydrates (C–S–H gel), and a small amount of needle-like ettringite distributed in pores and cracks characterize SEM-1 concrete’s microstructure, which is generally loose and porous. This microstructure inevitably impacts the concrete’s mechanical properties and impermeability.

The cementitious system of SEM-2 concrete comprises cement and fly ash. The resultant microstructure after the hydration reaction is denser than that of SEM-1, with significantly fewer pores and cracks. The hydration products are more integrated. The improvement in SEM-2 concrete’s microstructure results from the addition of fly ash, which enhances the cement’s hydration reaction while providing an effective filling effect and volcanic ash effect [56]. Fly ash reacts with cement’s hydration product Ca(OH)_2_ to generate more crystals to fill the pores and cracks, creating a denser microstructure and thereby improving the strength and durability of the concrete.

The cementitious materials of SEM-3 concrete include cement and CCCW. Although its apparent morphology resembles that of SEM-1, it has a reduced pore structure. Compared to SEM-1, SEM-3 displays improved overall integrity and compactness, further supporting the notion that adding CCCW can enhance concrete’s internal structure and, thus, its impermeability.

In the case of SEM-4 concrete, where the composite cementitious system consists of ordinary Portland cement, fly ash, and CCCW, the microstructural characteristics post-hydration reaction indicate significantly reduced crystal structure defects, thorough hydration reaction, and abundant C–S–H gel complexed into a whole. These characteristics suggest a significant improvement in structural compactness, demonstrating that the three cementitious materials have excellent compatibility and mutual coordination, collectively enhancing the microstructure of the concrete. This is also the main reason for the improvement in the macroscopic mechanical properties and impermeability of the concrete.

(2) Microscopic Morphological Characteristics of the Transition Zone between Fiber and Concrete Matrix at 28 Days of Age

**Figure 15:**
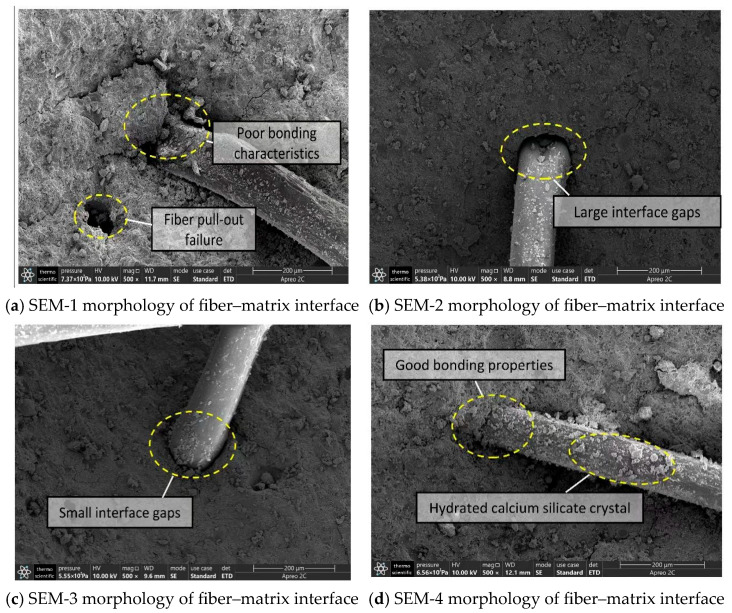
Microscopic morphology characteristics of the fiber–matrix interface area at a magnification of 500 times at 28 days of age.

Figure 15a–d presents the microscopic morphological characteristics of the bonding transition zone between the internal fibers and the matrix of the four concrete groups. The magnification is 500 times for electron microscopy scanning. The images show differences in the bonding characteristics between the concrete matrix formed by different composite cementitious systems and polypropylene fibers. In SEM-1 concrete, the phenomenon of fibers being pulled out occurred due to many matrix defects [57]. Consequently, the bonding strength between the fibers and the matrix was not high, resulting in large gaps at the bonding interface. In SEM-2 concrete, although the matrix is relatively smooth with fewer defects, large gaps in the interface transition zone between the fibers and the matrix still exist, indicating a lack of bonding strength between fly ash concrete and polypropylene fibers. This situation could prevent the fibers from fully exerting their reinforcing and toughening effects. In SEM-3 concrete, the gap in the bonding transition zone between the fibers and matrix is smaller, indicating an improvement in bonding strength [58]. The fibers appear to block the penetration of microcracks, explaining why polypropylene fibers can enhance the concrete’s impermeability. In SEM-4 concrete, the gaps in the transition zone between the fiber–matrix interface are further reduced, and the fiber surface is covered with hydrated calcium silicate crystals. This morphological feature can strengthen the synergistic force between the fibers and the concrete matrix, allowing them to fully exert their crack-resistant effect.

Through comparing and analyzing the microscopic morphological characteristics of the four concrete groups at 28 days of age, we found that concrete with only cement as the cementitious material has many internal defects, including poor crystal integrity, more pores and crack structures, and an incomplete hydration degree. The addition of fly ash or CCCW can somewhat improve the microstructure of the concrete matrix and the bonding strength between polypropylene fibers and the concrete matrix. When cement, fly ash, and CCCW are combined as cementitious materials for concrete, the improvement effect is most significant, and the three display good compatibility characteristics.

### 4.2. XRD Diffraction Phase Analysis

#### 4.2.1. XRD Diffraction Test Plan

The influence of fly ash and CCCW on the cement hydration reaction was evaluated through XRD diffraction tests after preparing four sets of concrete specimens. These four sets include the reference concrete, single fly ash concrete, single CCCW concrete, and composite fly ash and CCCW concrete. As polypropylene fibers mainly play a physical role within concrete and do not affect the cement hydration reaction, they are not added to these test pieces. The specimen numbers and proportions for the XRD diffraction test are presented in Table 18.

#### 4.2.2. XRD Diffraction Test Results and Analysis

**Figure 16:**
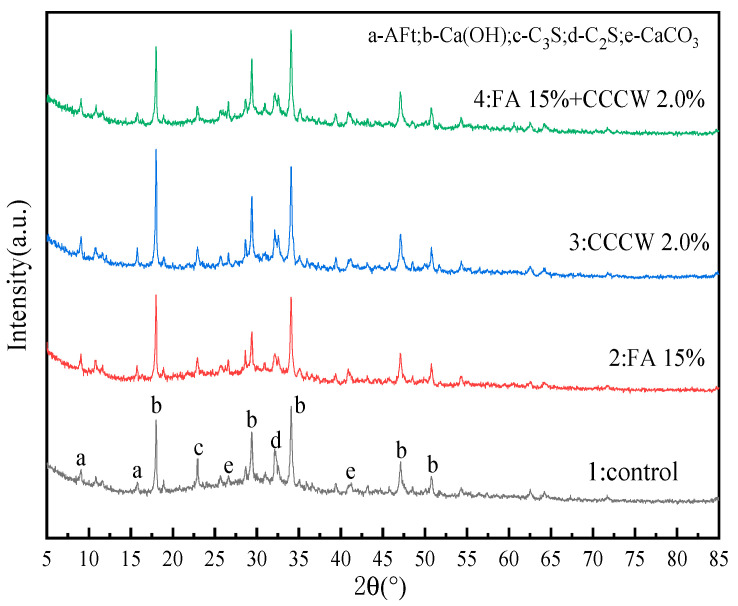
XRD spectrum of hydration products of concrete at 28 days of age.

The XRD diffraction patterns of the four concrete groups at 28 days of age can be obtained by analyzing and organizing the test data, as shown in Figure 16. The terms “control”, FA, and CCCW represent reference concrete, fly ash, and cementitious capillary crystalline waterproofing materials, respectively. A comparative analysis of the diffraction patterns of the four concrete groups revealed the influence of fly ash and CCCW on the cement hydration reaction.

From Figure 16, it can be seen that the single or combined addition of fly ash and CCCW does not affect the types of concrete hydration products, only influencing certain characteristic peak strengths. The phase qualitative analysis of the characteristic peaks shows that the main crystals in the concrete samples include Ca(OH)_2_, Ettringite Aft, CaCO_3_, and the non-hydrated cement components C_3_S and C_2_S. As the primary product of the cement hydration reaction—calcium silicate hydrate gel—is an amorphous phase [59,60], it cannot be characterized by diffraction patterns.

By comparing the spectra of No. 1 and No. 2, it is found that the diffraction peak intensity of Ca(OH)_2_ in the cement hydration products, after adding fly ash, slightly increases. This is because fly ash not only promotes the formation of gel-like hydration products but also acts as the activation center of Ca(OH)_2_ crystallization, enhancing the crystallinity of Ca(OH)_2_ and consequently increasing its characteristic peak strength [61]. The chemical equation of the cement hydration reaction shows that the content of the C–S–H gel and Ca(OH)_2_ are positively proportional and that C–S–H gel is an important source of concrete strength and durability [62]. In the case of fly ash replacing cement in equal amounts, the percentage of cement is reduced but the degree of hydration is higher, which indicates that fly ash can save the amount of cement while making the internal structure of concrete more dense and improving it. The characteristic peak strengths of C_3_S and C_2_S can also illustrate this phenomenon.

When comparing the No.1 and No.3 spectra, it is apparent that the addition of CCCW significantly strengthens the characteristic peak of Ca(OH)_2_. This suggests that CCCW can improve the crystallinity of cement hydration products, making the hydration reaction more complete and generating more hydration products. This could improve the strength and durability of concrete to some extent. By analyzing the No. 4 spectrum, it is found that, although the proportion of cement in the XRD-4 sample is the lowest, its Ca(OH)_2_ diffraction peak intensity is still slightly higher than in the No. 1 and No. 2 spectra. This strongly suggests that the composite addition of fly ash and CCCW does not interfere with the hydration process of cement but instead enhances the crystallinity of its hydration products.

## 5. Conclusions

This study employs an orthogonal experimental design to investigate the effects of various quantities of fly ash, polypropylene fibers, and CCCW on the mechanical properties and impermeability of concrete. This design determines the optimal composite dosage for each performance indicator, conceives a specific mixture ratio accordingly, and conducts scanning electron microscopy (SEM) tests and XRD tests on the concrete. The key findings are as follows:

(1) The relative impact of the three materials on concrete’s compressive strength is: fly ash > polypropylene fiber > CCCW. The optimal combination of these three components (A_2_B_1_C_2_) resulted in a compressive strength of 49.8 MPa, a 29.3% increase compared to standard C35 concrete;
(2) The influence of the three materials on the splitting tensile strength of the concrete is as follows: polypropylene fiber > CCCW > fly ash. The optimal combination for crack resistance (A_3_B_2_C_2_) resulted in a tensile strength of 6.31 MPa, representing a 56% improvement over the control concrete;
(3) The effect of the three materials on the concrete’s impermeability is: CCCW > polypropylene fiber > fly ash. The optimal combination for impermeability (A_1_B_2_C_2_) decreased permeability by 63.6% compared to the control concrete. Moreover, A_1_B_2_C_2_ also provided the optimal solution in the multi-indicator analysis;
(4) The 28-day-old control concrete exhibited numerous internal voids and crack defects and a low bonding strength between the polypropylene fibers and the concrete matrix. The incorporation of either fly ash or CCCW led to a substantial improvement in the concrete’s microstructure, resulting in a denser distribution of hydration products and improved fiber-to-matrix bonding characteristics;
(5) The addition of fly ash and CCCW effectively enhanced the cement hydration reaction and increased the crystallinity of the hydration products. The secondary hydration reaction of the fly ash and the complex precipitation reaction of the CCCW materials consumed a portion of the Ca(OH)_2_, which improved the structure of the aggregate interface transition layer, ultimately augmenting the concrete’s strength and durability.

## Figures and Tables

**Table 1:** Physical and mechanical performance indicators of cement.

Number	Requirement of Normal Consistency/%	Stability	Fineness/%	Setting Time/min		Flexural Strength/MPa		Compressive Strength/MPa	
P·O 42.5	26.7	Qualified	1.3	Initial set	Final set	3 d	28 d	3 d	28 d
				170	235	3.68	7.24	17.5	52.3

**Table 2:** Main performance indexes of cementitious capillary crystalline waterproofing materials.

Test Items		Technical Requirements	Test Results
Appearance		Homogeneous, no lumps	Homogeneous, no lumps
Water content/%		≤1.5	0.0
Fineness, 0.63 mm sieve residue/%		≤5.0	1.2
Chloride ion content/%		≤0.1	0.05
Water reduction rate/%		<8.0	7.0
Gas content/%		≤3.0 >−90 -	2.0 −75 -
Difference in setting time	Initial setting/min		
	Final coagulation/h		

**Table 3:** Performance indicators of polypropylene fibers.

Test Items	Technical Requirement	Detection Result
Tensile strength/MPa	350–537	469
Elongation at break/%	≤30	28.4
Elastic modulus/MPa	≥4000	4236
Density/g·cm^−3^	0.91	0.91
Diameter/mm	18–78	32.7
Melting point/°C	169	169

**Table 4:** Calculated Mix Proportion of C35 Benchmark Concrete.

Raw Material	Cement	Fine Aggregate	Coarse Aggregate	Water	Water-Reducing Agent
Consumption per cubic meter/kg·m^−3^	400	712	1112	176	0.8
	1	1.78	2.78	0.44	0.002

**Table 5:** C35 benchmark concrete test mix ratio.

Raw Material	Cement	Fine Aggregate	Coarse Aggregate	Water	Water-Reducing Agent
Consumption per cubic meter/kg·m^−3^	400	748	1076	176	0.8
	1	1.87	2.69	0.44	0.002

**Table 6:** Test factors–level table.

	Factor	A Fly Ash Content	B Fiber Content	C CCCW Content
Level				
1		15%	1.0 kg/m^3^	1.0%
2		20%	1.5 kg/m^3^	2.0%
3		25%	2.0 kg/m^3^	3.0%

Note: The proportion of fly ash and CCCW in the table refers to the percentage of total cementitious materials in the concrete mix proportion.

**Table 7:** Sample mix ratio.

Number	Water–Binder Ratio	Cement	Medium Sand	Gravel	Water	Water-Reducing Agent	Fly Ash	Fiber	CCCW
L-0	0.44	400	748	1076	176	0.8	0	0	0
L-1	0.44	336	748	1076	176	0.8	60	1.0	4.0
L-2	0.44	312	748	1076	176	0.8	80	1.5	8.0
L-3	0.44	288	748	1076	176	0.8	100	2.0	12.0
L-4	0.44	328	748	1076	176	0.8	60	1.5	12.0
L-5	0.44	316	748	1076	176	0.8	80	2.0	4.0
L-6	0.44	292	748	1076	176	0.8	100	1.0	8.0
L-7	0.44	332	748	1076	176	0.8	60	2.0	8.0
L-8	0.44	308	748	1076	176	0.8	80	1.0	12.0
L-9	0.44	296	748	1076	176	0.8	100	1.5	4.0

Note: The material usage in the table is kg/m^3^.

**Table 8:** Analysis of compressive strength range.

Test Piece Number	A Fly Ash Content/%	B Fiber Content/kg·m^3^	C CCCW Content/%	Compressive Strength/MPa
L1	15	1.0	1.0	40.7
L2	20	1.5	2.0	49.6
L3	25	2.0	3.0	38.4
L4	15	1.5	3.0	38.1
L5	20	2.0	1.0	45.7
L6	25	1.0	2.0	41.6
L7	15	2.0	2.0	38.2
L8	20	1.0	3.0	48.7
L9	25	1.5	1.0	38.7
K_1_	117	131	125.1	A > B > C
K_2_	144	126.4	129.4	
K_3_	118.7	122.3	125.2	
k_1_	39.0	43.7	41.7	
k_2_	48.0	42.1	43.1	
k_3_	39.6	40.8	41.7	
R	9.0	2.9	1.4	

Note: Taking factor A as an example, if the three tests with factor A level of ‘1’ are extracted, a set of tests can be obtained. In this group of experiments, A_1_ appeared three times, while the three levels of B and C appeared once, respectively. The data of this group of experiments and $K_{1}^{A}$, and the like, can be calculated as $K_{2}^{A}$, $K_{3}^{A}$.

**Table 9:** Analysis of variance for compressive strength.

Source of Variance	Sum of Squares of Variation	Degree of Freedom	Mean Square	F Value	*p* Value	Significance
A	152.4	2	76.2	66.8	0.01	Significant impact
B	12.6	2	6.3	5.5	0.15	Has a certain impact
C	4.0	2	2.0	1.8	0.36	No effect
Error	2.3	2	1.1			

**Table 10:** Analysis of the range of splitting tensile strength.

Test Piece Number	A Fly Ash Content/%	B Fiber Content/kg·m^3^	C CCCW Content/%	Tensile Strength/MPa
L1	15	1.0	1.0	4.59
L2	20	1.5	2.0	6.29
L3	25	2.0	3.0	4.99
L4	15	1.5	3.0	5.92
L5	20	2.0	1.0	4.26
L6	25	1.0	2.0	5.10
L7	15	2.0	2.0	4.85
L8	20	1.0	3.0	4.54
L9	25	1.5	1.0	5.86
K_1_	15.35	14.22	14.72	B > C > A
K_2_	15.09	18.07	16.24	
K_3_	15.95	14.11	15.44	
k_1_	5.1	4.7	4.9	
k_2_	5.0	6.0	5.4	
k_3_	5.3	4.7	5.1	
R	0.3	1.3	0.5	

Note: Taking factor A as an example, if the three tests with factor A level of ‘1’ are extracted, a set of tests can be obtained. In this group of experiments, A_1_ appeared three times, while the three levels of B and C appeared once, respectively. The data of this group of experiments and $K_{1}^{A}$, and the like, can be calculated as $K_{2}^{A}$, $K_{3}^{A}$.

**Table 11:** Analysis of variance of splitting tensile strength.

Source of Variance	Sum of Squares of Variation	Degree of Freedom	Mean Square	F Value	*p* Value	Significance
A	0.13	2	0.06	1.55	0.39	No effect
B	3.39	2	1.70	40.85	0.02	Significant impact
C	0.39	2	0.19	4.64	0.18	Has a certain impact
Error	0.08	2	0.04			

**Table 12:** Analysis of penetration height range.

Test Piece Number	A Fly Ash Content/%	B Fiber Content/kg·m^3^	C CCCW Content/%	Penetration Height/mm
L1	15	1.0	1.0	62
L2	20	1.5	2.0	46
L3	25	2.0	3.0	79
L4	15	1.5	3.0	58
L5	20	2.0	1.0	77
L6	25	1.0	2.0	42
L7	15	2.0	2.0	49
L8	20	1.0	3.0	78
L9	25	1.5	1.0	56
K_1_	169	182	195	C > B > A
K_2_	201	160	137	
K_3_	177	205	215	
k_1_	56.3	60.7	65.0	
k_2_	67.0	53.3	45.7	
k_3_	59.0	68.3	71.7	
R	10.7	15.0	26.0	

Note: Taking factor A as an example, if the three tests with factor A level of ‘1’ are extracted, a set of tests can be obtained. In this group of experiments, A_1_ appeared three times, while the three levels of B and C appeared once, respectively. The data of this group of experiments and $K_{1}^{A}$, and the like, can be calculated as $K_{2}^{A}$, $K_{3}^{A}$.

**Table 13:** Analysis of variance for penetration height.

Source of Variance	Sum of Squares of Variation	Degree of Freedom	Mean Square	F Value	*p* Value	Significance
A	184.9	2	92.4	10.95	0.08	Impact
B	337.6	2	168.8	19.99	0.05	Significant impact
C	1094.2	2	547.1	64.79	0.02	Significant impact
Error	16.9	2	8.4			

**Table 14:** Range analysis of multiple indicator problems.

Number	A	B	C	Test Indicators			Comprehensive Score
				1 *	2 **	3 **	
L1	15	1.0	1.0	40.7	4.59	88	251.8
L2	20	1.5	2.0	49.6	6.29	104	300.5
L3	25	2.0	3.0	38.4	4.99	71	209.2
L4	15	1.5	3.0	38.1	5.92	92	263.8
L5	20	2.0	1.0	45.7	4.26	73	216.0
L6	25	1.0	2.0	41.6	5.10	108	303.5
L7	15	2.0	2.0	38.2	4.85	101	283.7
L8	20	1.0	3.0	48.7	4.54	72	215.7
L9	25	1.5	1.0	38.7	5.86	94	269.0
K_1_	799.4	771.1	736.8	A: Fly ash content/%; B: Fiber content/kg·m^3^; C: CCCW content/%; 1 *: Compressive strength value/MPa; 2 **: Splitting tensile strength value/MPa; 3 **: 150—Penetration height/mm;			
K_2_	732.2	833.4	887.8				
K_3_	781.7	708.9	688.7				
k_1_	266.5	257.0	245.6				
k_2_	244.1	277.8	295.9				
k_3_	260.6	236.3	229.6				
R	22.4	41.5	66.4				

**Table 15:** Analysis of variance for multiple indicator problems.

Source of Variance	Sum of Squares of Variation	Degree of Freedom	Mean Square	F Value	*p* Value	Significance
A	807.84	2	403.92	9.71	0.09	Impact
B	2581.82	2	1290.91	31.02	0.03	Significant impact
C	7193.00	2	3596.50	86.42	0.01	Significant impact
Error	83.23	2	41.61			

**Table 16:** Optimal combination validation test results.

Factor Level Combination	Compressive Strength/MPa	Tensile Strength/MPa	Penetration Height/mm	Comprehensive Score
Benchmark concrete	38.5	4.03	121	101.8
A_2_B_1_C_2_	49.8	5.53	52	283.7
A_3_B_2_C_2_	41.3	6.31	47	293.9
A_1_B_2_C_2_	43.3	5.98	44	301.6

**Table 17:** Grouping and proportioning of scanning electron microscope specimens.

Number	Water–Binder Ratio	Cement	Grit	Stone	Water	Water-Reducing Agent	Fly Ash	Polypropylene Fiber	CCCW
SEM-1	0.44	400	748	1076	176	0.8	0	1.5	0
SEM-2	0.44	340	748	1076	176	0.8	60	1.5	0
SEM-3	0.44	392	748	1076	176	0.8	0	1.5	8.0
SEM-4	0.44	332	748	1076	176	0.8	60	1.5	8.0

Note: The material usage in the table is kg/m^3^.

**Table 18:** XRD specimen numbers and mix proportions.

Number	Water–Binder Ratio	Cement	Grit	Stone	Water	Water-Reducing Agent	Fly Ash	Polypropylene Fiber
XRD-1	0.44	400	748	1076	176	0.8	0	0
XRD-2	0.44	340	748	1076	176	0.8	60	0
XRD-3	0.44	392	748	1076	176	0.8	0	8.0
XRD-4	0.44	332	748	1076	176	0.8	60	8.0

Note: The material usage in the table is kg/m^3^.

## Data Availability

The data presented in this study are available on request from the corresponding author.
